# Current and Emerging Treatment Options for Pancreatic Cancer: A Comprehensive Review

**DOI:** 10.3390/jcm14041129

**Published:** 2025-02-10

**Authors:** Umar Hayat, Phillip S. Croce, Aseel Saadeh, Karna Desai, John Appiah, Sidrah Khan, Yakub I. Khan, Kishore Kumar, Ahmad Hanif

**Affiliations:** 1 Department of Internal Medicine, Geisinger Wyoming Valley Medical Center, Wilkes-Barre, PA 18711, USA; pcroce1@geisinger.edu (P.S.C.); kadesai@geisinger.edu (K.D.); jkappiah@geisinger.edu (J.A.); skhan14@geisinger.edu (S.K.); 2 Department of Internal Medicine, Geisinger Medical Center, Danville, PA 18711, USA; absaadeh@geisinger.edu; 3 Department of Internal Medicine, Division of Gastroenterology, Geisinger Wyoming Valley Medical Center, Wilkes-Barre, PA 18711, USA; ykhan1@geisinger.edu (Y.I.K.); kkumar@geisinger.edu (K.K.); 4 Department of Internal Medicine, Division of Hematology/Oncology, Geisinger Wyoming Valley Medical Center, Wilkes-Barre, PA 18711, USA; ahanif@geisinger.edu

**Keywords:** pancreatic ductal carcinoma (PDAC), neoadjuvant therapy, target therapy, immunotherapy, CAR-T

## Abstract

Pancreatic ductal adenocarcinoma (PDAC) is one of the leading causes of death worldwide, and its global burden has increased significantly over the past few years. The incidence of pancreatic cancer has also increased in the United States, and most of this increase is attributed to the population’s aging process in addition to the rise in the prevalence of risk factors such as obesity, diabetes, smoking, and alcohol intake. Most patients with pancreatic cancer present with advanced unresectable or metastatic disease. Only a few patients present at an early stage with localized disease, and a multidisciplinary approach is required to maximize survival and outcomes. The surgical approach is an option for localized disease, and surgery’s safety and efficacy have also been improved in recent years due to the increasing use of minimally invasive surgical techniques. Moreover, systematic chemotherapy has also been used and has had a significant impact on survival. More recently, neoadjuvant therapy has been used for pancreatic cancer along with radiation therapy, optimizing survival among those patients. Targeted therapies have been introduced based on genetic testing in metastatic pancreatic cancer and have shown promising results. Moreover, immune checkpoint inhibitors and targeted agents such as PARP inhibitors and vaccines have emerged with optimal results in terms of survival. To conclude, pancreatic cancer is considered a disease with poor long-term survival; however, recent developments in pharmacotherapy have changed its treatment and have improved outcomes with improved survival. Our review summarizes ongoing therapeutic options for local and metastatic pancreatic cancer. It also summarizes new state-of-the-art therapies that have emerged or are in trials, which can change the pancreatic cancer treatment perspective.

## 1. Introduction

Pancreatic ductal carcinoma (PDAC) is the third leading cause of cancer-related death in developed countries, including Europe and the United States [1,2]. Approximately 495,773 cases of pancreatic cancer were diagnosed worldwide in 2020 [2]. Furthermore, 66,440 new pancreatic cancer cases were diagnosed in 2024 in the US, with an estimated 51,750 deaths, and these trends have been increasing in recent years [3]. Moreover, pancreatic cancer constitutes approximately 8.5% of all cancer-related deaths in the USA. The overall 5-year survival rate (2014–2020) is only 12.8%, and this poor prognosis is associated with the aggressive local growth of this cancer and its early systemic spread [3]. Only 10–15% of patients present with the early stage disease, while 25–30% present with regional disease, and about 50–60% present with distant metastasis, contributing to the poor prognosis associated with this disease [3]. PDAC presentation in the advanced stage is attributable to the anatomical location of the pancreas, with cancer involving the superior mesenteric artery and veins early in the disease course, limiting the efficacy of currently available treatment options. Additionally, the molecular heterogeneity of cancer results in varying and suboptimal clinical responses to the best available conventional chemotherapy [4]. The treatment and prognosis of the PDAC also depend on the resectability of the tumor at the time of the presentation, as well as surgical expertise, as incomplete resection results in poor survival and quality of life [5]. These factors have led to the recent trend of neoadjuvant approaches for pancreatic cancer treatment [5].

Although PDAC is still a poorly understood disease, recent developments in the surgical field have improved survival rates for localized pancreatic disease. Similarly, systematic therapy has evolved with the growing interest in genomics and advancements in targeted therapies. [6] This review discusses the evidence behind current PDAC therapies and the novel treatment options in development to improve survival among these patients.

## 2. Current Treatment Options

### 2.1. Localized Pancreatic Cancer

Pancreatic cancer is one of the leading causes of cancer-related death in the United States, with most patients presenting with metastatic or unresectable disease [7]. About 10–15% of patients present with localized disease; in these cases, multidisciplinary management has proven necessary to optimize outcomes [8].

#### 2.1.1. Surgical Options

The NCCN guidelines classify localized pancreatic ductal adenocarcinoma (PDAC) according to resectability to help guide treatment decisions. Resectable PDAC is characterized by no significant contact with major arteries (celiac axis, SMA, or common hepatic artery) and minimal or no involvement of veins (SMV or PV), with ≤180° contact without vein contour irregularity. Borderline resectable PDAC is defined by tumor contact of ≤180° with the SMV/PV but with sufficient healthy vessels for reconstruction. Locally advanced or unresectable PDAC involves > 180° contact with the SMA or CA, involvement of the aorta, or unreconstructible SMV/PV due to tumor invasion or occlusion [9].

The choice of surgery may differ depending on the location of the tumor. Tumors located distal to the pancreatic head are usually amenable to a distal pancreatectomy; however, those located in the head and uncinate process require a pancreaticoduodenectomy (PD) for complete resection. PD was debuted in 1889 by the Italian Dr. Codivilla and was later fully established by Dr. Whipple in 1935 through his two-step description [10]. Surgical outcomes have improved in the last few decades with the availability of aggressive surgical techniques, including vascular resection and reconstruction [11]. In 1994, Gagner and Pomp reported the first laparoscopic PD (LPD) for treating chronic pancreatitis localized in the head of the pancreas in pancreas divisum [12]. Since then, the method has gained popularity, with recent studies showing similar complication rates, overall survival, and 5-year survival rates between LPD and PD, as well as shorter lengths of hospitalization in patients undergoing LPD. Similarly, robotic PD (RPD) has primarily observational evidence to justify its use [13,14]. However, these surgical procedures have been significantly associated with short-term morbidity and mortality. Among the most common complications related to PD are pancreatic fistula (an average incidence of 5–15%) and delayed gastric emptying (20%). Surgical outcomes for PDAC have improved in the past decades due to the increased expertise in surgical techniques, including vascular resection and reconstruction [15]. This leads to a higher survival rate of 20–30%. However, there are still higher recurrence rates associated with surgical treatments, likely due to the presence of micro-metastasis at the time of surgical intervention [14,16].

The NCCN and MD Anderson criteria for pancreatic cancer surgical resection are mainly similar, particularly in defining resectable tumors as those confined to the pancreas without central vascular involvement. However, they differ in some key areas. MD Anderson adopts a more lenient approach to vascular encasement, considering tumors with short-segment involvement or limited arterial encasement resectable if reconstruction is feasible. In contrast, NCCN requires no vascular involvement or minimal contact without irregularity. Both guidelines categorize tumors with lymph node involvement or distant metastasis as unresectable. Notably, MD Anderson has a broader definition of borderline resectability, accepting cases with potential for organ resection or vascular reconstruction, which NCCN considers unresectable. Overall, MD Anderson offers a more flexible stance on resectability, particularly regarding vascular involvement and adjacent organ encroachment.

Table 1 explains the surgical criteria for pancreatic cancer resection.

#### 2.1.2. Chemotherapy—Adjuvant

Even in those diagnosed with a localized, resectable tumor, the prognosis remains poor, with only a 20% 5-year survival rate following surgery alone, with this rate increasing to 30–40% with adjuvant chemotherapy [17,18]. Several trials over the past two decades have solidified the importance of adjuvant chemotherapy after the resection of PDAC, with this combination offering the only hope for long-term survival or cure in patients with nonmetastatic pancreatic cancer [18]. Based on the patient’s functional status, the recommendation is either for modified FOLFIRINOX (fluorouracil, oxaliplatin, irinotecan, leucovorin) for those with a high functional status or gemcitabine and capecitabine or gemcitabine alone for those with a low functional status. The CONKO-001 trial showed modestly improved median overall survival in patients treated with gemcitabine (22.8 months) compared to those who underwent observation (20.2 months) in the 6 months following surgical resection. Adding erlotinib to gemcitabine did not improve outcomes in an adjuvant setting [19]. The combination of adjuvant gemcitabine and cisplatin was evaluated in a phase II trial involving 22 patients that showed a median OS of 35.5 months. The combination was feasible, although a high percentage of patients (59%) experienced grade ¾ toxicity [20]. The landmark ESPAC-1 trial also showed the benefit of adjuvant 5FU/leucovorin with improvement in mOS from 14.0 months to 19.7 months [21]. The ESPAC-4 phase 3 trial showed an added survival benefit in those who received dual-agent therapy (gemcitabine plus capecitabine) over gemcitabine alone. The above results demonstrated a consistent pattern in adjuvant chemotherapy, a survival benefit of single-agent chemotherapy, and a further improvement in OS with the addition of capecitabine or cisplatin to gemcitabine. Another combination available in adjuvant settings is gemcitabine and nab-paclitaxel, which was evaluated in the APACT trial. The original analysis did not show a statistical difference, but an updated analysis in 2021 showed a significant difference in median overall survival with a longer follow-up (41.8 vs. 37.7 mo, *p* = 0.0091) [22].

In 2018, the PRODIGE-24 trial, which included patients with excellent functional status, showed a significant improvement in favorable survival in patients who received modified FOLFIRINOX (54.4 months) or gemcitabine alone (35 months) [23,24]. The higher-than-average median survival in the control group was likely reflective of the superior performance status of the patient population in this trial compared to others. Thus, using FOLFIRINOX remains limited to patients with an ECOG performance status of 0–1.

#### 2.1.3. Chemotherapy—Neoadjuvant

Neoadjuvant chemotherapy has gained popularity over the last decade, owing to several trials showing its effectiveness in borderline resectable pancreatic cancer (BRPC) and locally advanced pancreatic cancer (LAPC). When neoadjuvant chemotherapy is employed, patients should undergo restaging scans and then proceed to surgery if no disease progression is noted. Since radiographic response in pancreatic cancer does not correlate with pathological response, a lack of tumor shrinkage on imaging by itself should not preclude surgery in these patients. The NUPAT-01 trial showed encouraging outcomes with neoadjuvant therapy using either FOLFIRINOX or GemNabP in patients with borderline resectable pancreatic cancer with R0 resection rates of 67.4% and an mOS of 39.4 months. A multi-institutional trial by Jang et al. demonstrated improved margin-negative resection rates in patients with BRPC receiving neoadjuvant therapy [25]. Another analysis from National Cancer Database showed that patients receiving neoadjuvant treatment followed by resection for stage I/II pancreatic cancer had better OS rates than propensity score-matched patients who underwent upfront resection (mOS 26 months vs. 21 months, HR = 0.72, *p* < 0.01) [26]. Considering that a significant portion of patients cannot receive adjuvant systemic treatment due to operative morbidity, neoadjuvant therapy can increase the number of patients eligible for systemic treatment by potentially eradicating occult metastatic disease. However, its benefit in resectable PDAC continues to undergo investigation. A potential downfall of neoadjuvant therapy is the possibility of facilitating tumor progression in the setting of inadequate tumor response [25]. Important trials evaluating the benefit of neoadjuvant therapy in pancreatic cancer are listed below in Table 2.

#### 2.1.4. Radiation Therapy

The role of radiotherapy in PDAC is controversial, with the optimal regimen remaining unclear due to ongoing investigation. The PREOPANC trial found that neoadjuvant gemcitabine-based chemoradiotherapy improved overall survival compared with upfront surgery and adjuvant gemcitabine in patients with resectable and borderline resectable PDAC. The phase 2 ESPAC-5F study found that patients who received pre-operative chemoradiation had increased 1-year survival rates. Several other studies also suggested improved outcomes with neoadjuvant chemoradiation [28,29]. However, in the ALLIANCE trial, which compared treatment with neoadjuvant-modified FOLFIRINOX (mFOLFIRINOX) with or without hypofractionated radiation therapy, the effectiveness of radiotherapy could not be concluded, with no improvement in median overall survival noted [17,30]. ESPAC-1 trial also reported inferior outcomes with the addition of radiation to 5FU-based regimens in an adjuvant setting.

The LAP-07 trial evaluated the use of chemoradiation using capecitabine in patients with unresectable pancreatic cancer who did not progress after 4 months of gemcitabine chemotherapy. Chemoradiation did not improve OS compared to continuing gemcitabine for two more months. There was a small benefit in PFS with chemoradiation in this group, and thus, this approach can be considered if the patient desires to take a chemotherapy break.

In summary, the role of chemoradiation in locally advanced pancreatic cancer is not clear. It can be considered in the adjuvant setting after R1 resection and in the neoadjuvant setting for select patients after a multidisciplinary review. In the neoadjuvant setting, data from small studies and retrospective analyses show improved rates of R0 resection with chemoradiation; further information from randomized clinical trials is needed. However, in the neoadjuvant setting, the use of radiation with FOLFIRINOX is not recommended owing to negative results from the SWOG study [31].

#### 2.1.5. Maintenance Therapy

About 5–9% of patients with pancreatic cancer have germline or somatic mutations in the BRCA1 or BRCA2 gene. Data indicate a response to PARP inhibition in this subset of platinum-sensitive patients. The phase 3 POLO trial evaluated the role of maintenance olaparib and showed improved median progression-free survival but no difference in overall survival in interim analysis. Olaparib gained approval in the USA in December 2019 for patients with BRCA-mutated pancreatic cancer and stable disease after first-line chemotherapy, becoming the first biomarker-based targeted therapy approved for pancreatic cancer. This is an ongoing field of investigation, and the role of maintenance therapy remains unclear [17,32].

## 3. Unresected/Metastatic Pancreatic Cancer

Treatment options for advanced pancreatic cancer necessitate a strategic approach. Patients face a competing risk between locoregional and systemic progression, both of which impact overall prognosis. Approximately one-third of these patients succumb to local progression without any metastasis. Systemic therapy, either alone or in combination with locoregional treatment, has been utilized [33].

### 3.1. Surgical Therapeutic Options

The palliative management of pancreatic cancer focuses on relieving biliary and duodenal obstructions and controlling pain. Endoscopic stenting is preferred over surgical bypass for biliary obstruction due to its minimally invasive nature and quicker recovery. A review of 29 randomized trials confirmed stents as superior for palliation, with the choice of stent depending on prognosis: plastic stents (e.g., polyethylene) are preferred for patients with a life expectancy under six months due to their lower cost, while metal stents (e.g., Wallstents) are chosen for those with longer prognoses because they carry a lower risk of recurrent bile duct obstruction [34]. For duodenal obstruction, endoscopic stenting is ideal for patients with limited survival, whereas surgical gastrojejunostomy is considered for those with better overall status or combined obstructions. Pain associated with PDAC can be managed with celiac plexus blocks or thoracoscopic splanchnicectomy, especially when conventional analgesics are insufficient. Endoscopic options are typically favored for their tolerability and effectiveness, but the choice of intervention should be guided by the patient’s prognosis and overall condition [31,34].

### 3.2. Chemotherapy

Multiagent chemotherapy is the cornerstone of treatment for metastatic pancreatic ductal adenocarcinoma (PDAC).

Single-agent chemotherapy has been studied for metastatic cancer treatment. In a phase II randomized trial, the effect of the FIRGEM regimen, which includes the infusion of irinotecan delivered before and after the 5-FU/Leucovorin (FOLFIRI.3) and fixed-dose gemcitabine monotherapy, was assessed. The median progression-free survival was 6 months with FOLFIRI.3 and 3.4 months with gemcitabine (HR, 0.59; 95% CI, 0.38–0.90) [35]. Moreover, rates of hematological toxicity were higher in the combination therapy cohort. Moreover, FOLFIRINOX, compared to gemcitabine, yielded a dramatic improvement in the median progression-free survival (PFS) (6.4 months vs. 3.3 months, *p* < 0.001) and median overall survival (11.1 months vs. 6.8 months, *p* < 0.001) [35,36].

Several other combinations of gemcitabine have been explored, with some benefits observed from combination therapy. For example, the MPACT trial showed improved survival at 42 months among patients who received gemcitabine plus albumin-bound paclitaxel. Similarly, gemcitabine with 5FU, erlotinib, cisplatin, and capecitabine has shown few or no benefits for PDAC treatment [37,38,39,40].

The NAPOLI 3 trial began at the onset of the COVID-19 pandemic. In 2023, this multinational trial, conducted in 18 countries globally, marked a significant advancement in the treatment of metastatic pancreatic adenocarcinoma with the introduction of nalirifox, a newly favored regimen comprising liposomal irinotecan, fluorouracil, leucovorin, and oxaliplatin.

A recent randomized controlled trial demonstrated that nalirifox was more effective than gemnabp as the initial treatment for metastatic pancreatic ductal adenocarcinoma. The overall survival reported among the patients who received nalirifox was 11.1 months, compared to 9.2 months for those on gemnabp (HR = 0.83; *p* = 0.04). Moreover, there was a small difference in the adverse effect profile of both drugs (322 out of 370, or 87% for nalirifox vs. 86% for gemnabp); however, the patterns of toxicity varied, as nalirifox was associated with more gastrointestinal issues, whereas gemnabp had a higher incidence of hematologic toxicity. Treatment-related fatalities were recorded in six patients (2%) from the nalirifox group and eight patients (2%) from the gemnabp group [41].

Nichetti et al., in a systematic review and meta-analysis, compared three primary treatment regimens: nalirifox, folfirinox, and gemnabp. This highly anticipated review examined seven phase 3 clinical trials, encompassing 2581 patients (383 patients receiving nalirifox, 433 patients receiving folfirinox, and 1756 patients receiving gemnabp). Progression-free survival was found to be longer in patients treated with nalirifox (7.4 months [95% CI, 6.1–7.7]) and folfirinox (7.3 months [95% CI, 6.5–7.9]) than in those who received gemnabp (5.7 months [95% CI, 5.6–6.1]). Gemnabp yielded a shorter overall survival (OS) of 10.4 months (95% CI, 9.8–10.8) when compared to nalirifox (HR, 1.18 [95% CI, 1.00–1.39]; *p* = 0.05). In contrast, there was no significant difference in overall survival between folfirinox (11.7 months [95% CI, 10.4–13.0]) and nalirifox (11.1 months [95% CI, 10.1–12.3]; HR, 1.06 [95% CI, 0.81–1.39]; *p* = 0.65) [42].

Recent studies have shown that among patients with metastatic adenocarcinoma from a BRCA germline variant, the use of olaparib following initial platinum-based therapy enhances progression-free survival compared to the placebo (7.4 months vs. 3.8 months; hazard ratio for disease progression or death, 0.53; 95% confidence interval [CI], 0.35 to 0.82; *p* = 0.004) [23,43].

In 2023, the FDA approved the use of pembrolizumab, an immune checkpoint blocker, in patients with unresectable or metastatic microsatellite instability-high (MSI-H) or mismatch repair-deficient (dMMR) solid tumors that have progressed following previous treatment and who have no satisfactory alternative options. Only 1–2% of pancreatic cancers are MSI-H or dMMR, for which pembrolizumab is a valid option as a second line and beyond.

## 4. Pancreatic Cancer Emergent Therapies

### Target Therapy, Immunotherapy, and CAR-T

The current landscape of pancreatic therapies is ever-changing, and future/emerging therapies have gained momentum over the past several years. Below is a brief discussion regarding the above therapies and the overall future of pancreatic cancer therapy. 

Recent years have seen the approval of many targeted cancer therapies that can be used in any metastatic cancer with certain mutations for which first-line therapies have been exhausted. These include larotrectinib, entrectinib, and repotrectinib for NTRK-mutated cancers; pembrolizumab for high microsatellite instability (MSI-high), mismatch repair gene-deficient (dMMR), or high-tumor-mutation-burden (TMB) cancers; dabrafenib and trametinib for cancers with BRAF V600E mutation; selpercatinib for cancers with RET gene fusions; and fam-trastuzumab deruxtecan for cancers with an overexpression of HER2 oncoprotein. However, only 1–2% of pancreatic cancers exhibit MSI-high/dMMR, and the presence of other mutations discussed above is also very low.

Targeted therapy classically involves using identified germline or somatic mutations within cancer cells and creating a medication specifically targeting the affected pathway. One of the most mutated genes in pancreatic cancer is KRAS (involving the G12 codon). Unfortunately, the KRAS gene has been difficult to target. Sotorasib is an FDA-approved drug that targets the G12C codon and accounts for a small portion of the mutated gene. Currently, clinical trials are investigating the combination of sotorasib with other therapies [44]. Adagrasib is another targeted therapy for the G12C codon pathway currently being investigated. It has demonstrated encouraging results and is reportedly well tolerated [45]. Additional KRAS G12C targets are undergoing clinical trial investigation with minimal preliminary data [45].

While the above therapies appear to have some utility, G12C exhibits low expression in pancreatic cancers. G12D is the most frequently mutated subtype allele involving the KRAS pathway in pancreatic cancer. Two medications are currently being investigated. MRTX1133 is a G12D inhibitor undergoing phase I/II clinical trial testing. RMC-9805 is another G12D inhibitor in phase I evaluation. Target therapy for KRAS is primarily difficult due to the allelic distribution in the mutations. This has led the medical community to research inhibitors for multiple KRAS mutant alleles, known as pan-RAS inhibition. RMC-6236 is currently under clinical investigation in a phase I trial [46].

The above medications require further trials related to specific allele mutations with KRAS and demonstrate the need to target upstream and downstream pathways that affect KRAS mutations. Upstream targets such as SHP2 and SOS1 are involved in KRAS signaling activation. These targets use nucleotide exchange to help the KRAS pathway cycle between inactive and active states. SHP2 and SOS1 inhibitors are being developed and undergoing early phase clinical trials [3]. Downstream targeting involves specific KRAS pathways, such as RAF-MEK-ERK and P13K-AKT-mTOR [46,47].

In addition to KRAS signaling pathways, other pathways, such as tyrosine kinase signaling, have offered benefits in other cancer therapies. This pathway is actively being studied in pancreatic cancer as a beneficial avenue for cancer therapy. The EGFR inhibitor erlotinib has previously been used for metastatic pancreatic patients, with evidence demonstrating prolonged survival. Other EGFR inhibitors, such as nimotuzumab (humanized antibody), are currently being studied. Different strategies have been used to employ focal adhesion kinases, which are non-receptor tyrosine kinase inhibitors that delay tumor growth. Current studies are testing FAK inhibitors with chemotherapy to monitor the degree of anti-tumor response. Finally, Bruton’s tyrosine kinase inhibitors are also being evaluated in clinical trials with chemotherapy, such as the BTK inhibitors acalabrutinib and pembrolizumab [46,47].

Additional therapies target gene fusions in known RET, NRG1, and NTRK alterations. Selpercatinib, pralsetinib, and other next-generation inhibitors are currently undergoing clinical trials to evaluate the efficacy of using RET inhibitors. Zenocutuzumab and seribantumab are NRG1 inhibitors (affecting the downstream MAPK and P13K pathways) and are also undergoing clinical trial investigation [44,47].

Moreover, the MTAP/PRMT/CDKN2A is another novel pathway which is generating interest. This pathway involves the MTAP gene, specifically the deletion of the PRMT5 gene (involved in DNA methylation) on chromosome 9p21 found in some pancreatic cancers in MTAP-deficient cells. Furthermore, the inhibition of CDKN2A (a cyclin-dependent kinase that interacts with PRMT5) is also being investigated, and solid tumors have demonstrated a loss of MTAP and CDK2A genes in some PDAC patients [46].

In the past 5 years, PARP inhibitors have been investigated further for BRCA 1/2 mutations. PARP inhibitors are often used in maintenance therapy as nonchemotherapy treatment options. A phase III clinical trial for olaparib showed an improvement in progression-free survival, which later led to its approval by the FDA. Rucaparib is another maintenance therapy that targets PALB2-mutated pancreatic cancer and has shown beneficial results for progression-free survival in clinical trials. The NCCN recommends rucaparib for use in metastatic pancreatic cancer with germline mutations in BRCA1/2 or PALB2. PARP inhibitors continue to gain interest from the scientific community, and several clinical trials are underway [44]. Table 3 lists all the trials evaluating new strategies for pancreatic cancer management.

Immunotherapy has had debatable success in pancreatic cancer treatment. For instance, the evaluation of CD40 agonists has sparked some interest in studies investigating the response to pancreatic cancer when combined with/without chemotherapy. Furthermore, chimeric antigen receptor T-cell therapy has also been scrutinized, with minimal evidence demonstrating efficacy in treating pancreatic cancer. Several clinical trials are underway to explore CAR-T’s role further [45,46,47,48] (Table 3).

**Figure 1:**
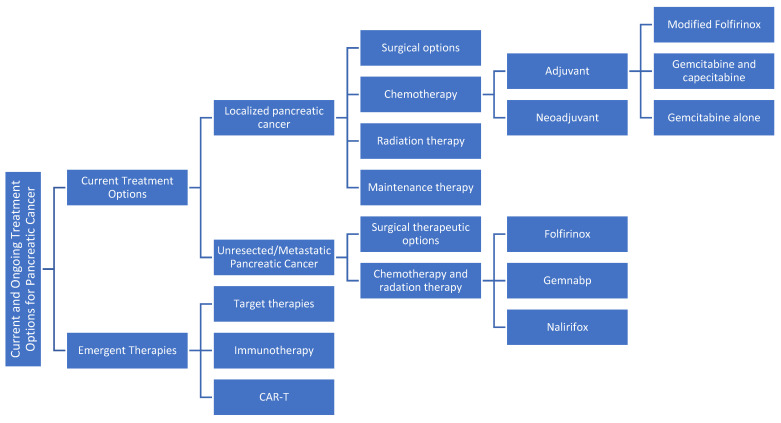
A flow chart of all the available therapeutic options for PDAC.

A flow chart of the manuscript, including all the available therapeutic options, is shown in Figure 1.

## 5. Discussion

In this review article, we have reviewed the current and ongoing therapeutic options for PDAC. The disease presentation determines the treatment option. Among the different surgical options, laparoscopic pancreatoduodenectomy (LPD) and open pancreatoduodenectomy (OPD) have been well-studied [39]. Two meta-analyses compared the LDP and OPD in terms of postoperative outcomes in 9144 and 15,278 patients with pancreatic cancer [49,50]. The reported rates of pancreatic fistulas and rates of mortality were similar between the two groups; however, R0 resections, harvested lymph nodes, overall survival rates, and 5-year survival rates were better in the LPD group than in the OPD group [49,50]. Other surgical options include the robotic pancreaticoduodenectomy (RPD) and the minimally invasive PD, and studies have shown no differences in the outcomes of both surgical methods [51]. Most patients present with disease recurrence due to metastasis, the liver being the most common organ for metastasis [52,53,54,55]. To minimize the risk of recurrence due to metastasis for those with local resectable disease undergoing surgical resection, neoadjuvant therapy has shown significant benefits in terms of survival, representing a substantial advancement in the treatment of PDAC. Surgical resection often requires extension into the vascular structure for successful R0 (complete resection) tumor resection. However, patients with locally advanced and metastatic disease on presentation should be treated with the definitive chemotherapy, which includes folfirinox, gemnabp, or gemcitabine + capecitabin with or without radiation. If the patient is responsive to initial chemotherapy, surgical resection can be considered for residual tumor removal. Pancreatic cancer with distal metastasis into different organs generally should not undergo resection [52,53,54,55]. However, certain advanced surgical centers around the world have attempted the surgical resection of PDAC for metastatic disease. Hackert et al. published a series on the resection of PDAC, in which they resected both liver and aortocaval lymph node metastasis in 128 patients. They reported a median survival of 12.5 months after resection. The overall 5-year survival was 5.9% and 7.0% after liver and nodal resection, respectively [56]. Wang et al. compared hepatic artery chemotherapy infusion therapy with adjuvant systematic chemotherapy among two groups of patients with PDAC metastasis in the liver and found that the 5-year disease-free probability was the same between the two groups; however, hepatic artery infusion group had better 5-year overall survival probability and hepatic metastasis-free survival [57]. With more data linked to identifying the role of genetic mutations, the future of pancreatic cancer therapy appears to relate to germline and somatic sequencing and exploiting targetable genetic mutations. Some target therapies are used against macrophages in the environment to help promote an anti-T-cell response to kill the target cancer cells. Additionally, there have been advances in immunotherapy with monoclonal antibodies and immune checkpoint inhibitors demonstrating efficacy in treating advanced and metastatic disease. Other avenues of therapy research have investigated the utility of stimulating dendritic cells and creating an adaptive immune response using cancer vaccines.

The gut microbiota is known to actively participate in the various physiological processes of the human body for survival and function. Recent murine model studies have shown that altering the gut microbiome may influence the development and progression of different cancers, immune responses, and treatment outcomes. New methods, including probiotics, prebiotics, fecal microbiota transplantation, dietary modifications, and antibiotics, may influence the gut microbiome by regulating the gut microbiota composition. This altered microbiome can significantly affect tumor progression, reshape the tumor environment, and enhance treatment outcomes. Further research is necessary to understand the particular manipulations in the gut microbiome and develop personalized PDAC therapies to treat this disease [58].

There are some limitations of our study. First, it is only a comprehensive review of the available trials on PDAC therapy, and there is a chance of missing the discussion of novel ongoing therapy trials, as there is a significant focus on research on this topic. Second, some personalized therapies which have shown equivocal results in the progression and treatment of PDAC have not been discussed in this review. Third, the eligibility criteria for each trial are not discussed in this review, so the generalizability of the chemotherapeutic options cannot be stratified to the common patient population. Lastly, the toxicity of the individual therapies has not been discussed in this study.

## 6. Conclusions

In summary, pancreatic cancer is an aggressive disease with grim survival outcomes. Advanced disease with metastasis at the time of presentation leaves few therapeutic choices. Specific new treatment options have become available with modest overall benefits. However, outcomes have continued to improve with surgical advancements and neoadjuvant therapy. New treatment avenues have been tested with variable efficacy, such as CAR-T cell immunotherapy, stimulating dendritic cells, and creating an adaptive immune response using cancer vaccines.

## Figures and Tables

**Table 1:** Criteria for pancreatic cancer surgical resection.

Criteria	Resectable (NCCN)	Resectable (MD Anderson Cancer Center)	Borderline Resectable (NCCN)	Borderline Resectable (MD Anderson Cancer Center)	Unresectable (NCCN)	Unresectable (MD Anderson Cancer Center)
**Tumor size**	Tumor confined to the pancreas, no involvement of major vessels	Tumor confined to the pancreas, no involvement of major vessels	Tumor involves major vessels but is amenable to resection with venous reconstruction	Tumor may involve local structures but with the potential for resection	Involvement of major vessels or distant metastasis, not amenable to resection	Extensive local invasion, distant metastasis, or unreconstructable vascular involvement
**Vascular involvement**	No involvement of major blood vessels (e.g., SMA, portal vein, hepatic artery), or ≤180° contact without vein contour irregularity	No involvement of major blood vessels (e.g., SMA, portal vein, hepatic artery), patent SMV-PV confluence	Vascular encasement of veins (portal/superior mesenteric) of >180° or ≤180° with contour irregularity or arterial involvement that can be resected or reconstructed	Tumor abutment of ≤180° of the circumference of SMA, short-segment encasement of the CHA or gastroduodenal artery, SMV, or SMV-PV, and patent vessel above and below	Encases or obstructs major blood vessels such as the SMA, celiac axis with > 180° contact and unreconstructable SMV/PV	Encasement of major vessels (SMA, portal vein, celiac axis) >180°, interface with the aorta, or unresectable venous occlusion
**Lymph node involvement**	No regional lymph node involvement (negative regional nodes)	No regional lymph node involvement (negative regional nodes)	Possible lymph node involvement, but resection may still be considered	Lymph node involvement that may be resectable	Extensive regional or distant lymph node involvement	Distant lymph node metastasis
**Distant metastasis**	No distant metastasis	No distant metastasis	No distant metastasis	No distant metastasis	Distant metastasis (e.g., liver, lungs)	Distant metastasis (e.g., liver, lungs)
**Adjacent organ involvement**	No invasion into adjacent organs like the duodenum, bile ducts, or stomach	No invasion into adjacent organs like the duodenum, bile ducts, or stomach	It may involve adjacent organs but is amenable to surgical resection	Involvement of adjacent organs but still potentially resectable	Invasion of adjacent organs such as the liver, stomach, or duodenum	Invasion of adjacent organs making resection not possible
**Overall prognosis**	Likely to benefit from curative surgery	Likely to benefit from curative surgery	High risk, but resection can still be performed with some degree of certainty	High risk, borderline, but resection may offer palliative outcomes	Poor prognosis, resection not an option	Poor prognosis, resection not an option

**Table 2:** Trials evaluating neoadjuvant therapy in pancreatic cancer management.

Trial Name	Regimen Used	Number of Study Participants	ECOG Performance Status	Median Survival	5-Year Survival
**PREOPANC-2**	FOLFIRINOX vs. gemcitabine-based chemoradiotherapy	368	0–1	Ongoing	Ongoing
**PREOPANC**	Gemcitabine-based chemoradiotherapy vs. upfront surgery	246	0–1	15.7 months (neoadjuvant) vs. 14.3 months (upfront surgery)	20.5% (neoadjuvant) vs. 6.5% (upfront surgery)
**NEONAX**	Perioperative gemcitabine + nab-paclitaxel vs. adjuvant gemcitabine + nab-paclitaxel	127	0–1	25.5 months (perioperative) vs. 16.7 months (adjuvant)	Not specified
**Macedo et al.** [27]	FOLFIRINOX vs. gemcitabine/nab-paclitaxel	274	0–3	33.4 vs. 30.7 months *p* = 0.804	30.3%

**Table 3:** List of clinical trials of emergent therapies for pancreatic cancer treatment.

Drug Categories	Drug Name	Trial Phase	Trial ID
**Target Therapy**			
Tyrosine kinase inhibitor	Fruquintinib	Phase 2	NCT05257122
	Anlotinib	Phase 2	NCT04718701
	Sunitinib	Phase 2	NCT06390826
	Penpulimab in combination with anlotinib	Phase 2	NCT06051851
Protein kinase inhibitor	Afatinib	Phase 2	NCT02465060
Vascular target photodynamic therapy	Padeliporfin	Phase 1	NCT05919238
panKRAS inhibitor	PF-07934040	Phase 1	NCT06447662
Antibody–drug conjugate	SOT102	Phase 1/2	NCT05525286
Mitochondrial metabolism inhibitor	CPI-613	Phase 1	NCT05325281
Monoclonal antibody	LYT-200	Phase 1/2	NCT04666688
MEK inhibitor	IMM-1-104	Phase 1/2	NCT05585320
	IMM-6-415	Phase 1/2	NCT06208124
KRAS inhibitor	QLC1101	Phase 1	NCT06403735
	LY4066434	Phase 1	NCT06607185
	RMC-6291 and RMC-6236	Phase 1	NCT06128551
	RMC-6291	Phase 1	NCT05462717
Recombinant fusion protein	JK08	Phase 1/2	NCT05620134
Bispecific antibody	FPI-2053	Phase 1	NCT06147037
**CAR-T**	iC9.CAR.B7-H3 T cells	Phase 1	NCT06158139
	CD276 CAR-T	Phase 1/2	NCT05143151
	CEA-targeted CAR-T	Phase 1/2	NCT06006390
	CEA-targeted CAR-T	Phase 1	NCT06126406
	CEA-targeted CAR-T	Phase 1	NCT06010862
	CEA-targeted CAR-T	Phase 1	NCT05415475
	CEA-targeted CAR-T	Phase 1	NCT05396300
	CEA-targeted CAR-T	Phase 1/2	NCT04348643
	C-13-60 cells	Phase 1	NCT06043466
	GPC3/mesothelin targeted	Phase 1	NCT06196294
	LY011	Phase 1	NCT04966143
	Mesothelin/GPC3/GUCY2C targeted	Phase 1	NCT05779917
	IX001	Phase 1	NCT06487377
	GB3010	Phase 1	NCT06054984
	NT-112	Phase 1	NCT06218914
	NT-175	Phase 1	NCT05877599
	Anti-claudin18.2	Phase 1	NCT05472857
	EPCAM	Phase 1	NCT05028933
	P-MUC1C-ALLO1	Phase 1	NCT05239143
**Vaccine**	KRAS peptide	Phase 1	NCT04117087
	KRAS peptide vaccine with poly-ICLC adjuvant	Phase 1	NCT05013216

## Data Availability

Not applicable.
